# Structural Polymorphism of Single pDNA Condensates Elicited by Cationic Block Polyelectrolytes

**DOI:** 10.3390/polym12071603

**Published:** 2020-07-19

**Authors:** Kensuke Osada

**Affiliations:** Quantum Medical Science Directorate, National Institutes for Quantum and Radiological Science and Technology (QST), Anagawa, Inage-ku, Chiba-shi, Chiba 263-8555, Japan; osada.kensuke@qst.go.jp; Tel.: +81-43-206-3274 (ext. 7939)

**Keywords:** DNA condensation, polyion complex, block copolymers, polyplex micelles, non-viral gene vector

## Abstract

DNA folding is a core phenomenon in genome packaging within a nucleus. Such a phenomenon is induced by polyelectrolyte complexation between anionic DNA and cationic proteins of histones. In this regard, complexes formed between DNA and cationic polyelectrolytes have been investigated as models to gain insight into genome packaging. Upon complexation, DNA undergoes folding to reduce its occupied volume, which often results in multi-complex associated aggregates. However, when cationic copolymers comprising a polycation block and a neutral hydrophilic polymer block are used instead, DNA undergoes folding as a single molecule within a spontaneously formed polyplex micelle (PM), thereby allowing the observation of the higher-order structures that DNA forms. The DNA complex forms polymorphic structures, including globular, rod-shaped, and ring-shaped (toroidal) structures. This review focuses on the polymorphism of DNA, particularly, to elucidate when, how, and why DNA organizes into these structures with cationic copolymers. The interactions between DNA and the copolymers, and the specific nature of DNA in rigidity; i.e., rigid but foldable, play significant roles in the observed polymorphism. Moreover, PMs serve as potential gene vectors for systemic application. The significance of the controlled DNA folding for such an application is addressed briefly in the last part.

## 1. Introduction

Human DNA, which is almost 2 m long, is packaged in a cellular nucleus and forms chromosomes approximately 10 μm in size through the integration of hierarchical higher-order structures. The higher-order structures dynamically change so that the transcription process can proceed in a controlled manner as necessary. How does DNA organize the higher-order structures and conduct dynamic structural changes? This is an essential question in the study of life sciences. To tackle this question, the nucleosome, a complex formed between individual DNA and a group of histones, can be highlighted, as it is the key basis for the organization of the hierarchical higher-order structures. A nucleosome is a polyion complex (PIC) formed between an individual negatively charged DNA and positively charged tails of histones rich in lysine and arginine residues. In this regard, PICs formed from DNA and cationic polyelectrolytes, or polycations, namely polyplexes have been investigated as simple models to gain insight into DNA folding from a materials science perspective [1,2,3,4,5,6]. The behavior of DNA after the polyion complexation is substantially different depends on DNA form whether it is long, short, double-stranded, or single-stranded. Double-stranded DNA is assumed as a rigid rod when it is shorter than 150 bp, which corresponds to its persistence length of 50 nm, whereas it behaves as a semi-flexible chain when it is sufficiently longer than the persistence length [7,8,9]. For latter case, DNA usually undergoes first-order transition by complexation with polycations changing its conformation from a worm-like expanded coil into a compact state. Shorter DNA than 150 base pair (bp) does not show such a volume transition by complexation with polycation. Polyion complexation with single-stranded DNA tends to result in micrometer-sized droplets in contrast to the double-stranded version that often results in precipitation [10,11,12]. Among these DNA forms, this review mainly deals with giant double-stranded DNA with a length of several thousand base pairs and focuses on its conformational changes upon polyion complexation.

We first discuss polyplexes formed from low- or high-molecular-weight polycations followed by those formed from polycations conjugated with hydrophilic neutral segments, such as poly(ethylene glycol) (PEG), namely block or graft copolymers. When copolymers are used, DNA undergoes folding as a single molecule encapsulated within a spontaneously formed polyplex micelle (PM) [13,14,15,16,17,18]. As a result, it can be observed that DNA is folded into several typical higher-order structures, including collapsed globular, rod-shaped, and ring-shaped (toroidal) structures [19]. This review attempts to gain insight into the structural polymorphism; how does the DNA strand arrange in these structures? why does DNA fold into such characteristic and various structures? and what are the conditions that differentiate these polymorphic structures? Besides, PMs possess potential structures as gene vectors to realize systemic gene therapy [14,15,16,17,20,21]. Significances of controlled DNA folding structures in the development of gene vectors and some critical issues residing there are addressed briefly.

## 2. DNA Condensation Induced by Low- or High-Molecular-Weight Polycations

DNA undergoes a large volume transition, called DNA condensation, when complexed with polycations to form polyplexes, which gives rise to decreasing its occupied volume by ~1000-fold. This volume transition is essential for genomic DNA packaging. Studies on DNA condensation often use phage DNA or plasmid DNA (pDNA) with several thousands to several tens of thousands of base pairs. These DNA are several millions in molecular weight and several micrometers in length. As a counterpart, typically, hexammine cobalt, spermine, or spermidine with charge numbers of 3 or 4 are used as low-molecular-weight polycations, while poly(L-lysine) (P(Lys)) or polyethyleneimine (PEI) are used as high-molecular-weight polycations. The size of high-molecular-weight polycations is usually several tens of thousand in molecular weight. Thus, DNA is a substantially large molecule when compared to polycations.

**Figure 1:**
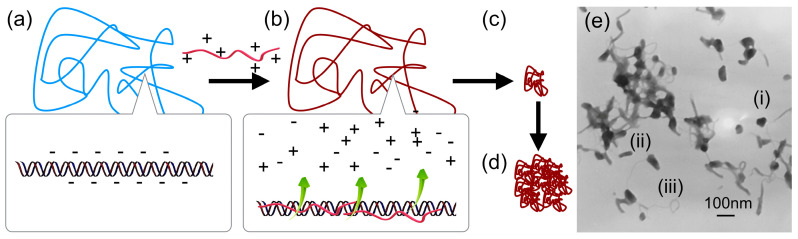
DNA condensation process induced by polycations. (**a**) DNA is hydrated to form coiled conformation. When complexed with polycations for polyion pairing (**b**), the polyplex undergoes volume transition by intra-polyplex association (**c**), followed by growth to large aggregates by inter-polyplex association (**d**). A representative TEM image of pDNA/P(Lys)_40_ polyplexes. (**e**)(i) globular, (**e**)(ii) rod-shape, and (**e**)(iii) toroid.

**Figure 2:**
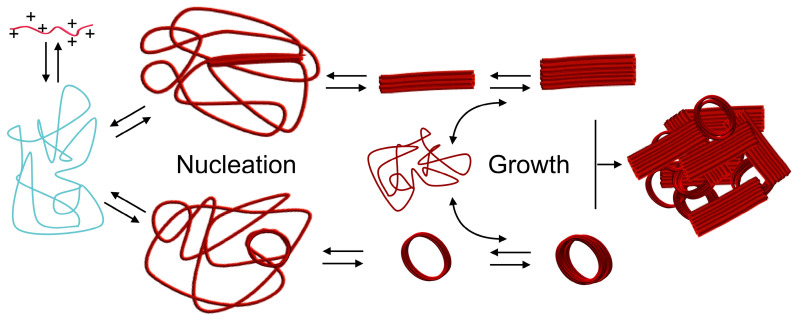
Possible scheme of DNA condensation process based on nucleation and growth for polyplexes of low molecular weight polycations.

The mechanism of DNA condensation is generally described as follows [6,22,23]. DNA in water assumes an expanded coil conformation due to the charge repulsion between phosphate groups residing along the chain (Figure 1a). When polycations are added, DNA and the polycations form a polyion pair for charge neutralization. This is driven by the entropy gained from the release of counterions bound to both polyelectrolytes into the bulk solution (Figure 1b) [24,25,26]. The charge-neutralized polyion pair undergoes chain rearrangement to reduce the unfavorable surface area in contact with water molecules, resulting in a volume transition into a compact form (Figure 1c). Subsequently, the polyplexes undergo secondary association to further reduce their surface area, grow into multimolecular aggregates, and ultimately precipitate (Figure 1d). In a specific case where the free energy in the expanded coiled state and the condensed state are equivalent, intramolecular phase segregation happens in a single DNA molecule with some domains coiled and others condensed [27,28,29,30]. The primary polyplexes have a hydrodynamic diameter of ~100 nm as measured using dynamic light scattering. The amount of polycations required to induce the volume transition largely depends on the number of their charges (degree of polymerization, DP). For low-molecular-weight polycations, the volume transition proceeds when an excess amount of polycations, with respect to the number of negative charges, is mixed with the DNA. In contrast, the volume transition proceeds near charge stoichiometry when high-molecular-weight polycations are used [31]. The morphology of polyplexes can be observed using transmission electron microscopy (TEM) and atomic force microscopy (AFM). The morphology tends to depend on the molecular weight of the polycation. Toroidal and rod-shaped morphologies are often observed in polyplexes formed from low-molecular-weight polycations [22,32]. The structure formation is explained by the model of nucleation and its growth [33,34]. The nucleation occurs in the equilibrium between association and dissociation (Figure 2). Once a nucleation site forms and is energetically favorable, the growth process proceeds through the incorporation of surrounding polyplexes to ultimately form rod-shaped or toroidal structures containing multimolecules of DNA [35,36,37]. The kinetics of the structure formation has been investigated in hexammine cobalt-based polyplexes. It was observed that polyplexes initially formed predominantly rod-shaped structures, which later transformed into toroidal structures over time [35]. From this result, it was presumed that the rod-shape structure is the kinetically favorable form, while the toroidal structure is the thermodynamically favorable form. Additionally, this dynamic structural change occurring over time suggested that these two structures are, to some extent, in equilibrium. In contrast, in the case of polyplexes formed using high-molecular-weight polycations, the globular structure was observed more often than the rod-shaped or toroidal structures. It was observed that when the DP of the polycation increased, the frequency of the globular structure formation increased. However, in many cases, polyplexes are observed as aggregates comprised of an unspecified number of DNA molecules. Hence, quantitative analysis of the higher-order structures of these polyplexes remains difficult [38,39]. A typical TEM image is presented in Figure 1e, which was taken from a sample formed using pDNA and P(Lys)_40_ (the subscript indicates the DP). It was presumed that the structure formation was kinetics-dominated because the polyion pairing between DNA and polycations became more polyvalent due to the increased DP in polycations as compared to the low-molecular-weight polycations. Hence, the equilibrium between association and dissociation was largely shifted toward association.

## 3. Single-DNA Condensation Regulated by Copolymers

**Figure 3:**
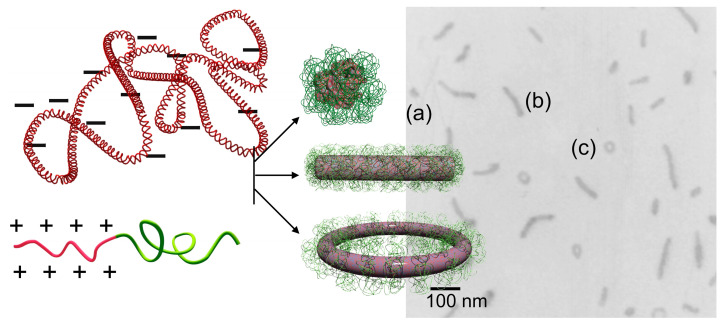
Single-DNA condensation by block copolymers. A representative TEM image showing the structural polymorphism; (**a**) globular, (**b**) rod-shaped, and (**c**) toroidal structures.

Higher-order structures formed from a single DNA can be obtained when polycations conjugated with neutral hydrophilic segments, namely block or graft copolymers, are used instead of the aforementioned “homo” polycations. This approach is important in investigating DNA folding because it allows the quantitative analysis of the single DNA condensates, as the parental chromosome comprises a single molecule of DNA. In this context, having condensates of individual molecules of DNA would be more appropriate than those comprising multiple DNA with an uncontrollable association number. The complexation between copolymers and individual DNA results in the formation of a PM with a core-shell structure in which the DNA is packaged in the core and the hydrophilic chains, typically poly(ethylene glycol) (PEG), form the shell [13,14,15,16,17]. The outer shell of the PM suppresses the secondary association of polyplexes to inhibit growth into multimolecular aggregates, thereby allowing DNA to undergo condensation as a single molecule inside a PM, even at charge stoichiometry. Notably, single DNA condensation is guaranteed when the complexation proceeds in dilute conditions. For example, in a complex of pDNA and PEG-*b*-P(Lys) block copolymer, single DNA packaging proceeds when the pDNA concentration is at most 0.35 mg/mL; otherwise, the polyplexes form a network involving multiple DNA [40]. This is because the formation of the PEG shell must be completed before surrounding DNA complexes collide due to translation motion. A typical TEM image of single pDNA condensates in PMs is shown in Figure 3, wherein globular, rod-shaped, and toroidal structures are observed to be coexistent. These structures are often found in PMs made from various copolymers [39,41,42,43,44,45,46,47,48].

Considering that the driving force of DNA condensation is to minimize the contact between the charge-neutralized polyplex and water molecules, it is reasonable to assume that the morphology after the condensation process would be spherical, as this shape has the lowest surface area. However, condensed DNA exhibits various structures instead of a spherical shape, as shown in Figure 3. Of note, the globular structure here terms the structure that appears round but is an indefinite shape, which distinguishes it from being spherical. Why is DNA condensed into such specific and various structures instead of a spherical structure? What are the conditions that differentiate these structures? How is DNA folded and arranged within these structures? These structural issues are discussed in the following sections.

### 3.1. Globular, Rod-Shaped, and Toroidal Structures

Among the three typical polymorphic structures, globular and rod-shaped structures are more often observed. The formation of these structures tends to depend on the DPs of the polycation and hydrophilic blocks of the copolymers. In PMs prepared using poly(2-(methacryloyloxyethyl phosphorylcholine))-*b*-poly(2-(dimethylamino)ethyl methacrylate) diblock copolymers (PMPC-*b*-PDMAEMA) with the DP of the PMPC block fixed at 30, the rod length decreased as the DP of the PDMAEMA block was increased from 10. Ultimately the globular dominated when the DP of the PDMAEMA block was greater than 60 [39]. In contrast, in PMs containing the same block copolymers with the DP of the PDMAEMA block fixed at 40, the rod length increased as the DP of the PMPC block was increased [39]. Similarly, in PMs made from PEG-*g*-Cysteine-P(Lys) with the DP of the P(Lys) block fixed at 30, the rod length increased when the molecular weight of PEG was increased [46]. The globular structure dominated when the DP of the polycation block was relatively higher and that of the hydrophilic block was relatively lower, such as in the cases of PEG_2kDa_-*b*-DMAEMA_100_ and PEG_2kDa_-*b*-P(Lys)_100_ [40,49]. Collectively, these observations indicated that as the DP of the polycation block increases, the rod length decreases and the formation of the globular structure becomes more frequent. In contrast, as the DP of the hydrophilic block increases, the rod length increases.

**Figure 4:**
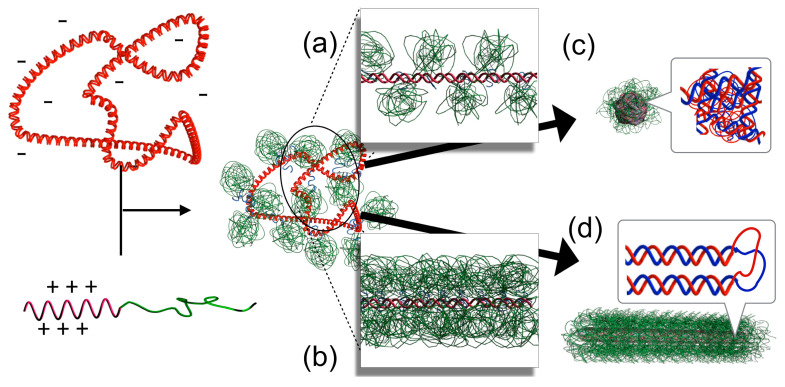
Schematic illustration of the critical role of PEG crowding in the process of pDNA condensation by block copolymers (**a**,**b**) and the integrity of double-stranded DNA in the condensates (**c**,**d**).

Based on this trend, a study that systematically modulated the molecular weight of PEG and the DP of P(Lys) in PEG-*b*-P(Lys) block copolymers discovered the factors involved in differentiating the formation of the rod-shaped and globular structures. During the DNA condensation process, polyion pairing first occurs between DNA and the polycations. Then, rearrangement of the complexed chains proceeds to reduce the surface area in contact with water molecules. In the case of copolymers, the second process proceeds with PEG attached to pDNA. Due to the large occupied volume of PEG, PEG likely interferes with the rearrangement process depending on the amount and the size of the PEG tethered to DNA. Thus, it would be necessary to consider the contribution of PEG to the copolymer-mediated condensation process. It is important to remark the correlation between the DP of the polycation and the number of copolymers associating with DNA. For a polycation block with a DP of at least 10, the number of copolymers that bind to a DNA corresponds to the amount needed to neutralize the negative charges of DNA. For example, for DNA of 5000 bp, the number of copolymers bound would be 500, 200, and 100 for cationic blocks with DPs of 20, 50, and 100, respectively. Accordingly, the number of PEG chains tethered to DNA increases as the DP of the polycation block decreases. As a result, the molecular weight of PEG and the DP of P(Lys) regulate the size and number of PEG bound to DNA, respectively, giving rise to a parameter by which the extent of interference of PEG in the rearrangement process can be controlled. Based on this correlation, the crowding of PEG tethered to DNA at the polyion pairing stage before undergoing condensation is estimated in terms of overlapping. The correlation with the crowding of PEG and the resulting condensed DNA structures demonstrated that the crowding of PEG on DNA at the polyion pairing stage regulated the pathways of chain rearrangement to form either globular or rod-shaped structures. It was revealed that the globular structure was preferentially formed when the tethered PEG chains did not overlap with one another prior to condensation (Figure 4a), whereas the rod-shaped structure was preferentially formed when the PEG crowding was dense enough to allow overlapping (Figure 4b) [40]. These results demonstrated the critical role of PEG in the regulation of the condensed DNA structures. As such, DNA could condense into globular structures, a more favorable shape in terms of the surface area, as long as the PEG chains permit the instantaneous transition; otherwise, the formation of the rod-shaped structure is promoted.

Pertaining to the trend associated with the rod-length, the presence of PEG also plays a critical role in determining the rod length. An energetic description of the rod-shaped structure is proposed to mechanistically explain the trend observed in PMs made from PEG-*b*-P(Lys) [50]. For rod-shaped structures, unfavorable interfacial free energy (*E_surface_*) is developed at the interface between the polyplex core and water, which results in the reduction of the rod length (*l*). However, the reduction of the rod length (d*l*) is opposed by the rigidity of the bundled DNA packed in the core with modulus *G*. Accordingly, the free energy for DNA compaction is represented by

d*F_compaction, DNA_* = *Gl*d*l* − d*E_surface_*. (1)

The reduction of the rod length forces the surrounding PEG chains into a narrower space in the shell, which generates repulsive elastic interactions due to the entropy loss of the resulting conformation (d*S_conf,PEG_*). Moreover, the osmotic pressure (Π) increases with increasing PEG crowding. Thus, the free energy for anti-compaction due to PEG is represented by

d*F_anti-compaction,PEG_* = Π(d*V_occ,PEG_*) − *T*(d*S_conf,PEG_*), (2)

where *V_occ,PEG_* and *T* represent the number-average occupied volume of PEG and temperature, respectively. For example, PMs prepared from P(Lys) with lower DPs contain a higher number of PEG chains on the shell, which tend to elongate the rod due to the increased PEG steric repulsion. A long rod, however, has a more unfavorable *E_surface_* compared with a short rod; consequently, a long rod develops higher free energy for compaction. As a result, the longer rod structure has higher PEG crowding compared with the shorter rod to balance the energy. The PEG crowding analysis showed that PMs made from PEG_12kDa_-*b*-P(Lys) with P(Lys) DPs of 70 and 20 had reduced tethering density (RTD) values of 2.6 and 5.2, respectively, indicating that the latter PM had a higher PEG density. The RTD values suggest that in the former PM, PEG adopts a mushroom conformation, while in the latter, PEG adopts an upward squeezed conformation. The observations were verified by observing the PEG height of the PM using cryo-TEM [50]. According to the energetic description, larger compaction energy develops in a rod structure that has a more hydrophobic surface. In this case, it is expected that the rod length would be shorter, and the PEG density would increase to balance the increased compaction energy. In fact, the rod-length of PMs prepared from block copolymers with hydrophobic cholesteryl groups attached to the end of the polycation block were observed to be shorter. Moreover, the PEG density of such PMs was higher than that of PMs without the cholesteryl group, despite the DPs of the PEG and polycation blocks being comparable, thereby supporting the proposed energetic description [51].

The energetic description was further examined through an experiment using a PEG-cleavable PM prepared from PEG_12kDa_-acetal-P(Lys)_19_ block copolymers in which the acetal linker connecting PEG and P(Lys) blocks was readily cleaved in an acidic environment [52]. The original rod-shaped structure changed to a globular structure upon incubating the PM in acidic media to release the PEG blocks from the PM. This observation verified the critical role of PEG in preventing the rod structure from becoming globular, which is the more favorable shape in terms of the *E_surface_*. Concomitantly, this study revealed the effect of bundled DNA rigidity on the rod shape. The rod length remained unchanged until PEG crowding decreased to a critical concentration, at which point the structure abruptly changed to globular instead of gradual decreasing in the rod length. This observation provides clear evidence of the contribution of the rigidity of bundled DNA in sustaining the rod-shaped structure. As the contribution of PEG continuously decreased upon detachment, an alternative contribution was present to sustain the rod-shaped structure until the point of transition. Notably, the critical concentration corresponded to the point at which PEG crowding between neighboring chains shifted from overlapped to non-overlapped. This study demonstrated that the rod-shaped structure is sustained by the synergistic contributions of PEG and DNA rigidity, but this structure collapses into a globular structure when these synergistic contributions are unable to maintain the necessary conditions. The scheme of the second-order transition from rod-shaped to globular suggests that these structures are in distinguishable phases. Ultimately, the structural studies that clarified the critical conditions needed to differentiate the formation of rod-shaped or globular structures, along with the energetic description of the rod-shaped structure, provide a general scheme for the formation of PMs irrespective of the polymers used [39,41,42,43,44,45,46,47,48].

**Figure 5:**
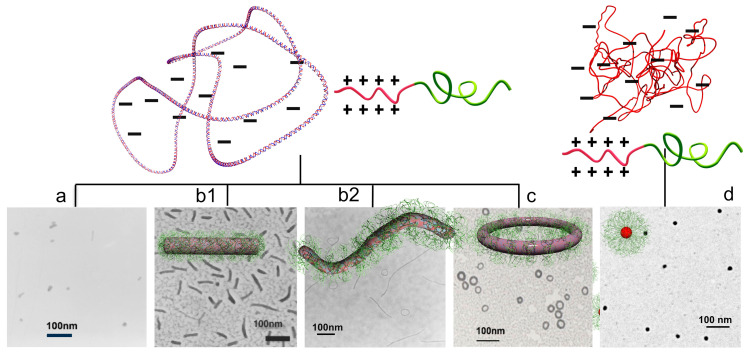
Control of higher-order structures of single pDNA elicited by block copolymers into globular-collapsing (**a**), rod-folding with folded more (**b1**) or less (**b2**), and ring-spooling (**c**). Single-strand DNA is folded into spherical (**d**).

The toroid is another characteristic structure that is observed less often but always in coexistence with the rod-shaped and globular structures [39,41,44,46,47,48,53]. The frequency of toroid formation does not significantly change upon modulating the DPs of PEG and polycation blocks. However, it was revealed that the salt strength strongly influenced the probability of toroid formation. A study in which NaCl concentration was modulated during the preparation of PMs using PEG_12k_-*b*-poly{N’-[N-(2- aminoethyl)-2-aminoethyl]aspartamide}_61_ (PEG-*b*-P(Asp(DET)) found that the rod-shaped structure was selectively formed when pDNA and the block copolymers were mixed in the absence of salt (Figure 5b). However, the frequency of the formation of the rod-shaped structure decreased and that of the toroidal structure increased when the PM was prepared in the presence of NaCl. Moreover, the toroidal structure was selectively formed when the PM was prepared in the presence of 600 mM NaCl (Figure 5c) [54].

The formed structures were stable, and no structural changes were observed over time, indicating that these structures were trapped at a local minimum in the energetic diagram and the energy barrier for the structural transformation is sufficiently higher than the thermal fluctuation.

This is in contrast with the polyplexes formed using low-molecular-weight polycations that occur in structural transformations in equilibrium. However, structural transformation occurred in PMs when the salt concentration, which affects the electrostatic interaction between DNA and the polycations, was changed after the PMs had already been prepared. In this case, the change from rod-shaped to toroidal was observed, but the reverse was not [54], suggesting that the toroidal structure is more thermodynamically stable than the rod-shaped structure, which is consistent with previous observations on DNA/hexamine cobalt polyplexes [35].

Ultimately, it is possible to selectively prepare globular, rod-shaped, or toroidal structures by regulating the interaction between pDNA and copolymers through the PEG steric repulsion modulatable by varying the DPs of the PEG or polycation blocks and the salt strength, as shown in Figure 5. Besides, there are various interaction modes for the polyion pairing, such as charge density along polycations, geometrical matching between positive charges on polycations and negative charges on DNA, solvent polarity, etc., and these affect the subsequent folding processes and the formed structures. For example, peptides octamer consisting of four positive charges of lysine residue (K) and four neutral residues of serine (S) elicited the formation of different higher-order structures depending on their sequences despite that the number of positive charges were identical. The peptides with their positive charges evenly distributed along the chain, KSKSKSKSKS, bound to DNA stronger and resulted in disordered globular structure while those placed at one side similar to a block arrangement, KKKKSSSS, bound to DNA weaker and resulted in ordered structures of rod-shape and toroid [55].

While the conditions for the different higher-order structure formation have been clear, an underlying mechanism still needs further study for clarification. Yet, granted the concept of nucleation and its growth for structure formation (Figure 2) [33,34], a possible mechanism may be considered as follows. To begin with, the higher-order structures of PM did not change to other higher-order structures unless the salt strength was changed as mentioned above [54], meaning that the structure has been destined in nucleation process. The first process of nucleation is the polyion pairing between DNA and polycations. This is regulated by the competition between the counterions condensed around the DNA strands and polycations, and proceeds when the entropy gain attained by the counterion release to bulk solution is more favored [24,25,26]. The polyion paired-DNA subsequently undergoes volume transition in order to reduce its contact area with water molecules. The DNA tends to form a globular shape for pursuing smaller surface area; however, the immediate transition is interfered when the PEG chains associated on the DNA strands present excess (Figure 4b). This interference may afford circumstances for the polyion-paired DNA to prepare a stable nucleus, eventually leading to folding of the DNA into a rod-shape, which is a highly ordered structure with quantized lengths and with hexagonal packing, most likely, as discussed in the next Section 3.2 [56]. In contrast, when abundant salt presents, the entropy gain by the counterion release will not be substantially high to promote the immediate polyion pairing [5]. In this case, enthalpy favorability between the charge-shielded polyelectrolytes might serve as an alternative interaction mode for promoting the polyion pairing. Furthermore, DNA becomes slightly flexible as evidenced by the reduced persistence length from 59 to 46 nm as the NaCl concentration increased from 2.57 to 1000 mM [57]. Such circumstances overall might afford a chance for DNA to prepare a more stable nucleus that leads to toroid-spooling. Noteworthy, toroid is thermodynamically more stable structure as compared to rod structures [35,54], which is consistent to the general acknowledgement that the structure formed via a slower process results in thermodynamically more stable structure.

### 3.2. Arrangements of DNA Strands in Rod-Shaped and Toroidal PMs

**Figure 6:**
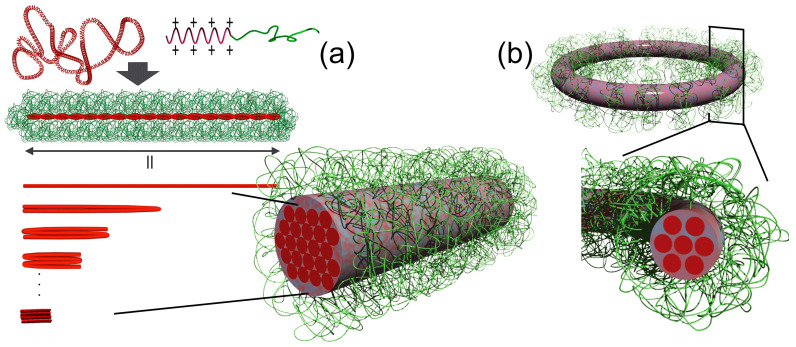
Specific folding scheme of pDNA induced by block copolymers. (**a**) A pDNA is folded into bundled-rod structure by quantized folding. (**b**) A pDNA is spooled into toroid. The toroid with 6-spooled pDNA contains 7 packed DNAs within the cross-section.

It is important to know how DNA is arranged within these polymorphic structures after elucidating the conditions needed to differentiate such structures. An interesting mode was found in the rod-shaped structure. The rod-length of PMs prepared from pDNA and PEG_12k_-*b*-P(Lys)_17_ exhibited a discrete distribution instead of a statistical distribution based on the analysis of TEM images. The lengths corresponded to multiples of 1/2(*n* + 1) of the contour length of pDNA folded *n* times, revealing a quantized folding rule in the pDNA condensate as shown in Figure 6a [56]. Thus, the rod-shaped structure is a bundle of folded pDNA consisting of 2(*n* + 1) numbers of DNA packed in the orthogonal cross-section. Notably, lateral packing is energetically favorable for rigid chains, such as DNA. Furthermore, lateral packing is more accomplished if the ends of the DNA strand, a cause for defects in perfect packing, are positioned at the ends of the rod. The quantized folding scheme thus allowed the energetically favorable arrangement of DNA. This folding scheme has been observed in rod-shaped PMs regardless of the PEG molecular weight, DP of the P(Lys) block, and species of polymers and pDNAs, indicating the generality of this folding scheme in rod-shaped PMs [47,48,58,59]. In contrast, toroidal structures formed in 600 mM NaCl solutions had a unimodal distribution in size (Figure 5c), suggesting the presence of a favorable scheme in pDNA spooling. The circumference measured from TEM images corresponded to pDNA spooled six times. This indicated that seven strands of DNA are packed in the orthogonal cross section of the toroidal structure. Interestingly, seven is the critical number required to form a hexagonal lattice as illustrated in Figure 6b [54]. This fact illustrates that lateral packing is an essential factor for the DNA arrangement in the condensates. Notably, cryo-TEM technique found hexagonal packing of 22 bp-short DNAs as a bundle in PMs [60] as well as phage DNA packaged in its capsid [61,62]. The hexagonal packing was also proved by small angle X-ray scattering (SAXS) technique in lipoplexes [63] as well as in polyplexes from spermine, P(Lys), P(L-arginine), and branched/linear PEI [64,65]. These observations overall indicate that DNA inherently prefer hexagonal packing. 

### 3.3. Folding Mechanism of DNA in PMs and Their Structural Polymorphism

Apart from the regulated folding schemes of pDNA, there is an apparent inconsistency in the structures and the intrinsic rigidity of DNA. DNA is assumed as a semiflexible chain in long-range order; however, it is assumed as a rigid rod in the local range shorter than the persistence length, ~50 nm, which corresponds to ~150 bp [7,8,9]. Then, the globular structure, which is smaller than the persistence length, and the rod-shaped structure, which accompanies back folding of DNA at the rod ends, cannot be explained. It is presumed that DNA in polyplexes might be slightly flexible as indicated by the reduced persistence length of DNA whose charges are compensated by Mg^2+^ (~44 nm) [8]. Granted this, the formation of these structures is still difficult to explain. Therefore, there may be a specific folding mechanism to adopt these structures. This was studied using S1 nuclease, which specifically cleaves single-stranded DNA. The gel electrophoresis results after the S1 nuclease treatment presented a smear pattern for globular pDNA [40], a distinct pattern for rod-shaped pDNA wherein the fragment lengths corresponding exactly to multiples of the rod length [45,56,58], and no cleavage for toroidal pDNA [54]. These fragmentation patterns indicated that the double-stranded structure was dissociated at non-specific sites in globular samples (Figure 4c), at localized sites at the rod ends of the rod-shaped samples (Figure 4d), and no dissociation remaining intact over the entire region in toroidal samples. Based on the distinct integrity of the double-stranded structure, a mechanism of rigid DNA folding was proposed; DNA undergoes double-strand dissociation to achieve back folding to adopt the rod-shaped formation, while dissociation occurs randomly in the globular structure. Importantly, unlike double-stranded DNA, single-stranded DNA is flexible with a persistent length of a few nanometers [7,9,66]; therefore, DNA folding takes place easily. Notably, this folding mechanism of DNA may find an answer to the pioneering observation that condensed DNA is fragmented by the *Neurospora cressa,* a single-strand DNA-specific endonuclease [67]. It is worth to emphasize that the smear pattern for globular samples indicated that the DNA was not completely digested but maintained the double-stranded structure partially intact. In this case, it is thought that such remaining double-stranded DNA portions interfere with the structure becoming spherical, presumably accounting for the formation of globular, instead of spherical, structures. In other words, a flexible DNA should form a spherical structure upon condensation. Consistently, polyplexes prepared from a flexible single-stranded DNA, which was obtained by dissociating double-stranded DNA via heat melting, presented only spherical structures upon being complexed with block copolymer, as shown in Figure 5d [68], and with homo-P(Lys) as well [69]. In such a way, the apparent inconsistency between the higher-order structures of polyplexes and the DNA rigidity could be explained. Presumably, such specific nature of DNA that is basically rigid but foldable may be relevant to the emergence of various higher-order structures. Of note, a simple rigid chain generally adopts ring-spooling to form a toroidal structure, and a flexible chain readily adopts a spherical structure.

## 4. PMs as Potential Gene Vectors

Precise control of protein expression levels plays a crucial role in maintaining homeostasis in the body. This indicates that proteins can be potent medicines to tackle various diseases. Administration of proteins is thus straightforward. However, it is difficult to maintain the injected proteins in the targeted tissue for a long time. Moreover, it is also difficult to manufacture and purify proteins for therapeutic purpose in a large scale due to cost. In this regard, an alternative approach has been considered: the administration of the source of the proteins, i.e., the DNA encoding the corresponding proteins, to the cells of interest for transcription and translation. This approach, namely the in-body production of medicines, is attractive because the medicines would exist at the site of interest as long as the transferred DNA exists inside the nucleus. Moreover, the medicines can be distributed to the whole body if secretory proteins are utilized. However, DNA is subject to digestion immediately upon being injected into the bloodstream. Additionally, DNA is incapable of penetrating the cellular membrane. Therefore, gene vectors that can transport DNA into nucleus of the cell and then promote transcription therein are necessary to realize this approach. In particular, gene vectors that are applicable via intravenous injection are desired because they allow minimally invasive administration without surgery, which enables frequent injection and the delivery to various cells via blood networks system that cover almost the entire body. Viruses are excellent gene vectors; however, there have disadvantages such as carcinogenicity and immunogenicity, the loadable size of the gene due to the limitations of the virus capsid, and the suitability in systemic application as viruses are eliminated by the inherent defense mechanism of the body. PMs possess several appealing properties due to their structure [14,15,16,20,21]. First, the size and the core-shell architecture of PMs are similar to those of viruses, which are approximately 100 nm in size and have a structure, wherein genomic DNA is condensed in the core and the capsid forms a shell. Second, PMs can package DNA without size restrictions into the core compartment. Third, the surrounding PEG shell can prevent the adsorption of opsonin proteins, which facilitate recognition and elimination from the bloodstream via the reticuloendothelial systems (RES), as well as the access of nucleases [70], thereby affording viability for systemic application [71]. In fact, PMs from PEG_10kDa_-*g*-P(Lys)_30_ have proceeded to clinical evaluations in humans for the gene therapy of cystic fibrosis in epithelia through intranasal administration as the first polymer-based formulation [72,73]. However, attempts at systemic application, wherein the structural features of PMs would be key advantages, still face challenges due to differences between the requirements of cellular, local, and systemic applications [74,75,76]. PMs need appropriate structures and functions to overcome the harsh biological conditions encountered throughout the delivery process, from the intravenous administration to the ultimate transcriptional processes at the target cell nucleus. The versatility of molecular designs afforded by polymer chemistry makes it possible to incorporate such required structures and functions into PMs. Efforts to promote PMs have invested into improving their structural stability [42,77,78,79] to circumvent disintegration due to shear stress in the bloodstream [80] and the polyion exchange reaction that potentially occurs when PMs encounter negatively charged polysaccharides, such as glycosaminoglycans (GAGs) that are abundantly present on the cell membrane and particularly in the glomerular basement membrane of the kidney [81,82]. Other efforts include the denser coverage of PEG to avoid RES capture [50,51], installation of hydrophobic palisade under the PEG shell to block the access of nucleases and GAGs to the packaged DNA in the core [47,48], attachment of targeting ligand molecules [83,84], and introduction of functionalities to control intracellular trafficking, such as endosomal escape wherein DNA is subject to enzymatic degradation [85,86,87,88].

A particular important issue among them is the longevity in bloodstream. Delivery of gene vectors, or widely nanomedicines, to tumor is basically mediated by “enhanced permeability and retention (EPR) effect”, which is based on the high permeability of the malignant vasculature to macromolecules and the impaired lymphatic drainage in tumor [89]. The extent of tumor accumulation by the EPR effect is currently controversial due to its heterogeneity in patient-to-patient and even in the lesions in the same patient [90,91,92]; nevertheless, the exerted therapeutic effect by several clinically used nanomedicines, such as Doxil, Abraxane, or antibody−drug conjugates, which use this route, demonstrates certain availability of the EPR effect [93]. In any case, the prolonged blood circulation is an indispensable basic property for gene vectors to increase the chance of tumor accumulation. Note that this requirement also applies to “active targeting” strategies mediated by the specific interaction between cellular receptors and targeting ligands because the extravasation first comes before the ligand utilization, unless vascular targeting strategy. It is widely accepted that PEGylation is effective for attaining prolonged blood circulation ability wherein the density is an issue. It has been reported that increasing the PEG density of PMs to the extent that could prevent serum protein adsorption; i.e., *L*/2*R*_g_ < 0.47, where *L* is the distance between tethering PEG chains and *R*_g_ is the radius of gyration of a PEG [94] resulted in an improved blood circulation profile that was considered as avoiding Kupffer cell-mediated clearance [50]. Such PMs after applying cross-linking could have achieved 99.9% retention in bloodstream per circulation in mice; in other word, only 0.1% of PMs were eliminated from bloodstream per cycle [95]. Nevertheless, this is still a large difference compared with polymeric micelles loading drugs that can circulate days with 99.99%/cycle [95]. The precise elimination mechanisms residing in this difference is still unknown. Nonetheless, one possible mechanism has recently been raised from a direct observation of PM circulation in liver using an intravital confocal laser scanning microscopy. The cross-linked PMs with the PEG density exceeding the critical limit, thus thought to be capable of circumventing the Kupffer cell-mediated clearance, were entrapped at the sinusoidal wall in the liver by time [96], indicating the sinusoidal wall is a marked elimination mechanism even after circumventing the Kupffer cell capture. Noteworthy, this entrapment was prevented by preinjecting two-armed PEG-conjugated oligo P(Lys) block copolymer for transient coating of the sinusoidal wall. This is intriguing because it allowed for preventing the sinusoidal clearance not only of PMs but also of viral gene vectors, AAV, thereby boosting their gene transfection efficiency in their target. Another important issue of gene vectors is the size. The smaller-sized vectors are afforded more chance of extravasation, and therefore accumulate more at the tumor. This is particularly important for targeting stroma-rich tumors, such as pancreatic tumors, which restrict the penetration of nanomedicines with sizes larger than 50 nm [97]. It should be noted that this size limit is critical for gene vectors because of the persistence length of DNA (~ 50 nm). Indeed, rod-shaped PMs showed accumulation at the periphery of the tumor stroma but not in the tumor nest [88]. In contrast, PMs prepared from single-stranded DNA, which was obtained by melting the pDNA, thereby circumventing the rigidity issue of double-stranded DNA, could accumulate deep in the tumor nest, exerted gene expression, and elicited antitumor effect in the pancreatic tumor model [68]. Aside from the structural issues, it is important to understand the binding behavior of polymers to DNA to properly evaluate the performance of the developed gene vectors. As previously mentioned, copolymers will not bind to DNA in ratios that would exceed the number of negative charges of DNA, unless the copolymers have strong hydrophobic groups, such as cholesteryl group [98]. Accordingly, if prepared under conditions wherein the number of charges of the polycation greatly exceeds the number of negative charges of the DNA, the solution would consist of charge stoichiometric polyplexes and an excess fraction of free polycations that do not participate in the polyplex formation. Polyplexes prepared in this way have sometimes shown enhanced transfection efficiency compared with those prepared at charge stoichiometry. However, it should be noted that the enhancement is derived from the free polymers and not from the improved performance of the polyplex itself. Such free polymer assistance may be useful in cellular transfection but cannot be expected for systemic application due to the different pharmacokinetics and biodistribution between polymers and PMs. More importantly, in contrast with their positive effects, free polymers are causes of cytotoxicity [99]. Therefore, the development of PMs with no free polymers is important [88].

It should be noted that study to devote the DNA folding plays a crucial role in these attempts because it directly affects the particle size, PEG density, arrangement of DNA strands within a PM, and also verifies the presence of free polymers. These factors eventually regulate the efficacy of the PM in the delivery process, such as the retention in blood [50,51,68,89,97,100] and the selection of cellular uptake pathways [78,101], the final transcription efficiency [40,55,58], as well as the safety issue [88].

There are many issues to be outlined in the development of gene vectors. However, further discussion is refrained here since the main focus of this review is on DNA folding. There are several informative review papers for gene delivery [95,102,103,104,105,106]. Notably, once a capable PM has been developed, it will not discriminate the genes to be loaded. Such pluripotency, similar to the relationship between hardware and software, is one of the appeals in non-viral gene therapy for tackling various intractable diseases.

## 5. Summary and Outlook

A PM allows single DNA folding. As a result, it was revealed that individual DNA inherently forms higher-order structures, such as rod-folding and ring-spooling, and otherwise results in the collapsing into globular that occurs when the condensation process instantaneously proceeds. The formation of these polymorphic structures can be controlled by regulating the interaction between DNA and the copolymers via the salt strength and the extent of steric hindrance of the hydrophilic block, which can be controlled by varying the DPs of the hydrophilic and cationic blocks in the copolymers. Specific characteristics of double-stranded DNA being inherently rigid but foldable may be relevant to the emergence of the polymorphism. It is worth noting that these formed structures persist over time but are capable of transforming when the electrostatic interaction between DNA and polycations is modulated by salts. This means that the higher-order structures are dynamically convertible.

DNA assembly with polycations is recently utilized in the field of DNA nanotechnology, or DNA origami technology. This technology is attractive because it allows for designing versatile higher-order three-dimensional structures precisely in nanoscales. However, their applications, particularly for biomedical use, have been hampered due to the inherent limitations that DNA origamis require the high content of Mg^2+^ ions typically with 10 to 20 mM [107], and moreover, DNA is not stable in physiological environment since it is spontaneously digested by nucleases [108]. To meet these issues, complexation with polycations is shown to be useful [109,110]. It is worthy to note here that complexation may induce collapsing of the origami structures because of the inevitable tendency of condensation and the charge neutralized complexes likely cause inter-origami aggregation as this review dealt with. Therefore, careful selection of polycations is indispensable. Particularly to the latter case, use of block copolymers could ensure a single origami coating as is the case of polyplex micelles [111,112].

As another approach, DNA assembly has recently been highlighted with relevance to membraneless organelles presenting in cellular or nuclear compartments, which are thought to play many important roles for organizing biochemical reactions [113,114,115,116,117]. They are an assembly from DNA or RNA and cationic polyelectrolytes but form as a result of liquid-liquid phase separation (LLPS). The assembly is observed as a micrometer-sized liquid droplet under optical microscopes, which is distinct from polyplexes, which are observed as solid precipitates under the optical microscopes if they are large enough to be observed as the cases of those formed with homo-polycations. Such droplets are typically formed from single-stranded oligo DNA or RNA in the presence of salt, whereas the double-stranded alternatives tend to precipitate under the same condition [10,11,12,118]. Notably, it was observed that oligo double-stranded DNA, approximately 20 bp, melted once incorporated in droplets [119]. It is considered that distinct characters between single- and double-stranded DNA, such as rigidity and charge density, associate with these distinguished properties. Double-stranded DNA can also form a droplet when the salt concentration is increased. In such a case, the formation of liquid crystalline ordering is found in the droplets [12], which is attributable to the inherent tendency of double-stranded DNA to pack laterally, as observed in polyplexes formed with various cationic compounds [60,61,62,63,64,65]. Intriguingly, such ordering was shown to be relevant with biological functions. In DNA assembly formed with antimicrobial peptides, the spacing between aligned DNAs determined the inflammatory responses to bacterial infection [120,121,122]. Besides polyion complexes, it has been known that DNA condensation occurs by neutral water-soluble polymers, such as PEG [28,67], and even by anionic polymers, such as poly(aspartic acid) and poly(glutamic acid) [123,124,125], which is accounted by the depletion attraction force developed between DNA molecules.

It should be important to note that in the nuclear compartment there are not only cationic substances but also various neutral and anionic substances present. DNA interacts with all of them and accomplishes its genome packaging, changes its higher-order structures dynamically, and ultimately generates its intrinsic functions. Of particular interest is that these processes take place in the macromolecular crowding circumstances in controlled manner [126,127]. It is a long way to understand these processes on a materials science basis, but at least such dynamic motion of higher-order structures of biomacromolecular assembly are permitted to take place in “liquid” phase. Therefore, understanding of higher-order structures of DNA assembly and their dynamics in liquid phase will be a next challenge.
